# A strange Evans syndrome: a case report

**DOI:** 10.4076/1757-1626-2-8001

**Published:** 2009-06-03

**Authors:** Julie Le Scanff, Stéphane Durupt, François Bailly, Agnès Rode, Pascal Sève

**Affiliations:** 1 Department of Internal Medicine, Hospices Civils de Lyon, Hotel Dieu, 1 place de l'Hôpital69288 Lyon Cedex 02, France and University Claude Bernard Lyon 1, LyonFrance; 2 Department of Internal Medicine, Châlon sur Saône Hospital71321 Chalon sur SaôneFrance; 3 Department of Hepatogastroenterology, Hospices Civils de Lyon, Hotel Dieu, 1 place de l'Hôpital69288 Lyon Cedex 02, France and University Claude Bernard Lyon 1, LyonFrance; 4 Department of Radiology, Hospices Civils de Lyon, Croix Rousse Hospital103, Grande-Rue de la Croix Rousse 69317 Lyon Cedex 04France

## Abstract

Hepatic angiosarcoma is a rare malignant vascular tumor, which accounts for up to 2% of all primary liver tumors. The most frequent symptoms on presentation are weight loss, weakness and abdominal pain. Diagnosis of diffuse hepatic angiosarcoma can be challenging. We report an original case of diffuse liver angiosarcoma revealed by haematological abnormalities initially diagnosed as an Evans syndrome. Anaemia and thrombocytopenia are rarely the first manifestations of this pathology. They are explained by combination of several mechanisms. Diagnosis of diffuse liver angiosarcoma can be extremely difficult and physicians should be aware of these presentation.

## Case presentation

A 47-year-old Caucasian man was referred to our department for investigation of an anemia and thrombocytopenia in November 2005. He had been in his usual good health until three months prior to his hospitalization when he started to suffer from fatigue and progressive loss of 15 kilograms. He worked in the aviation business and he had traveled several times in Africa up to 1989. He did not smoke or consume alcohol.

The patient had macrocytic anemia and thrombocytopenia. The lactate dehydrogenase was 796 U/l and reticulocytes 260 G/l. Haptoglobin and ferritin levels were low. Hemolysis work-up, including direct and indirect Coombs' tests was negative. No schizocyte and fibrin split product were found. Myelogram showed no pathologic medullar infiltration. His liver function tests revealed a mixed cholestatic/hepatitic abnormality. He had a low serum albumin and elevated prothrombine time. Factor V was 67% and activated partial thromboplastin time was normal. Evaluation was negative for viral hepatitis, hemochromatosis and Wilson's disease. The results of blood tests including alpha fetoprotein, fibrinogene level and autoantibodies were normal. Abdominal ultrasonography revealed enlarged homogenous liver, splenomegaly and moderate amount of ascites. There were signs of portal hypertension in gastroscopy. A steroid therapy was administrated for one month in hypothesis of Evans syndrome unsuccessfully.

**Figure 1.**
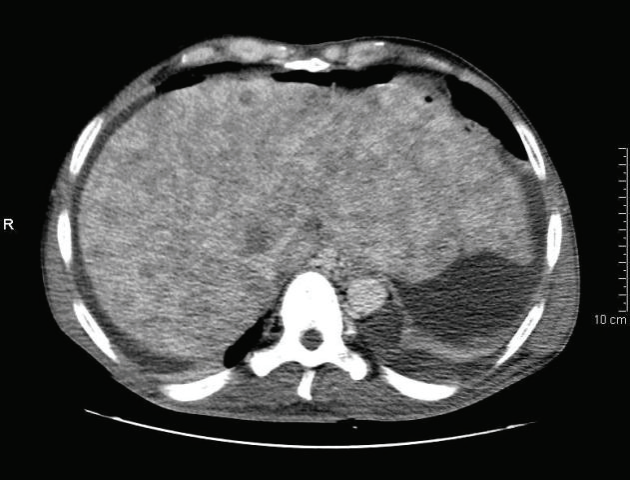
First abdominal computed tomography of our patient showing ascites and diffuse heterogeneous liver nodules, filled with contrast-like agent.

Physic status worsened and denutrition, jaundice, hepatosplenomegaly appeared. Ascites increased progressively. The patient was then referred to our department. Abdominal computed tomography (CT) showed ascites, splenomegaly and heterogeneous liver nodules, filled with contrast-like agent (Figure 1). Ascites puncture did not isolate malignant cells. Bone marrow examination was not contributory. Infectious disease such as tuberculosis, leishmaniasis, HIV and intracellular germs infections were eliminated. A first transjugular liver biopsy showed a centrolobular lesion with an appearance of myeloid metaplasia but interpretation was difficult because of the small size of the biopsy. The patient underwent then an exploratory laparotomy. Splenic histology was normal and the liver biopsy was not contributive since hemorrhagic. A new abdominal CT showed an increase of the liver nodules size. A second transjugular liver biopsy was then performed and histologic examination and immunohistochemistry were consistent with primary hepatic angiosarcoma. We did not start any active treatment because patient's conditions were poor. He died three months after initial presentation in hepatic failure.

Hepatic angiosarcoma is a rare malignant vascular tumor, which accounts for up to 2% of all primary liver tumors. Aetiologic factors, absent in our case, are exposure to thorium dioxide, polyvinyl chloride and arsenic. The most frequent symptoms on presentation are weight loss, weakness and abdominal pain [1]. Physical findings include ascites, hepatomegaly and jaundice. It is unusual that liver angiosarcoma is revealed by haematological abnormalities. [2]. Anaemia and thrombocytopenia may be related to microangiopathic haemolytic anemia, systemic disseminated coagulation, bleeding due to portal hypertension or hemoperitoneum, hypersplenism or, as in our case, combination of several mechanisms [1]: low ferritin and haptoglobin levels with no signs of chronic bleeding in gastroscopy or CT led to the diagnosis of intravascular hemolysis associated with portal hypertension. Classical CT findings are single or multiples hypoattenuating masses with heterogeneous enhancement on contrast-enhanced CT [1-3]. More rarely, diffuse liver angiosarcoma may appear as pseudo-peliosis with diffusely infiltrating micronodules, filled with contrast-like agent [3]. In these cases, diagnosis of diffuse liver angiosarcoma can be extremely difficult and liver biopsy has been reported as treacherous and non diagnostic [1]. The prognosis for patients with primary angiosarcoma of the liver is very poor with a median survival reported to be around 6 months [4].

## Conclusion

In summary, diagnosis of diffuse hepatic angiosarcoma can be challenging. Physicians should be aware of this presentation including haematological disorders and liver micronodules.

## Abbreviation

CT: Computed tomography
